# Placental growth factor testing to assess women with suspected pre-eclampsia: a multicentre, pragmatic, stepped-wedge cluster-randomised controlled trial

**DOI:** 10.1016/S0140-6736(18)33212-4

**Published:** 2019-05-04

**Authors:** Kate E Duhig, Jenny Myers, Paul T Seed, Jenie Sparkes, Jessica Lowe, Rachael M Hunter, Andrew H Shennan, Lucy C Chappell, PARROT trial group, Rachna Bahl, Gabrielle Bambridge, Sonia Barnfield, Jo Ficquet, Carolyn Gill, Joanna Girling, Kate Harding, Asma Khalil, Andrew Sharp, Nigel Simpson, Derek Tuffnell

**Affiliations:** a Department of Women and Children's Health, School of Life Course Sciences, King's College London, London, UK; b Division of Developmental Biology and Medicine, University of Manchester, Manchester, UK; c Research Department of Primary Care and Population Health, University College London, London, UK

## Abstract

**Background:**

Previous prospective cohort studies have shown that angiogenic factors have a high diagnostic accuracy in women with suspected pre-eclampsia, but we remain uncertain of the effectiveness of these tests in a real-world setting. We therefore aimed to determine whether knowledge of the circulating concentration of placental growth factor (PlGF), an angiogenic factor, integrated with a clinical management algorithm, decreased the time for clinicians to make a diagnosis in women with suspected pre-eclampsia, and whether this approach reduced subsequent maternal or perinatal adverse outcomes.

**Methods:**

We did a multicentre, pragmatic, stepped-wedge cluster-randomised controlled trial in 11 maternity units in the UK, which were each responsible for 3000–9000 deliveries per year. Women aged 18 years and older who presented with suspected pre-eclampsia between 20 weeks and 0 days of gestation and 36 weeks and 6 days of gestation, with a live, singleton fetus were invited to participate by the clinical research team. Suspected pre-eclampsia was defined as new-onset or worsening of existing hypertension, dipstick proteinuria, epigastric or right upper-quadrant pain, headache with visual disturbances, fetal growth restriction, or abnormal maternal blood tests that were suggestive of disease (such as thrombocytopenia or hepatic or renal dysfunction). Women were approached individually, they consented for study inclusion, and they were asked to give blood samples. We randomly allocated the maternity units, representing the clusters, to blocks. Blocks represented an intervention initiation time, which occurred at equally spaced 6-week intervals throughout the trial. At the start of the trial, all units had usual care (in which PlGF measurements were also taken but were concealed from clinicians and women). At the initiation time of each successive block, a site began to use the intervention (in which the circulating PlGF measurement was revealed and a clinical management algorithm was used). Enrolment of women continued for the duration of the blocks either to concealed PlGF testing, or after implementation, to revealed PlGF testing. The primary outcome was the time from presentation with suspected pre-eclampsia to documented pre-eclampsia in women enrolled in the trial who received a diagnosis of pre-eclampsia by their treating clinicians. This trial is registered with ISRCTN, number 16842031.

**Findings:**

Between June 13, 2016, and Oct 27, 2017, we enrolled and assessed 1035 women with suspected pre-eclampsia. 12 (1%) women were found to be ineligible. Of the 1023 eligible women, 576 (56%) women were assigned to the intervention (revealed testing) group, and 447 (44%) women were assigned to receive usual care with additional concealed testing (concealed testing group). Three (1%) women in the revealed testing group were lost to follow-up, so 573 (99%) women in this group were included in the analyses. One (<1%) woman in the concealed testing group withdrew consent to follow-up data collection, so 446 (>99%) women in this group were included in the analyses. The median time to pre-eclampsia diagnosis was 4·1 days with concealed testing versus 1·9 days with revealed testing (time ratio 0·36, 95% CI 0·15–0·87; p=0·027). Maternal severe adverse outcomes were reported in 24 (5%) of 447 women in the concealed testing group versus 22 (4%) of 573 women in the revealed testing group (adjusted odds ratio 0·32, 95% CI 0·11–0·96; p=0·043), but there was no evidence of a difference in perinatal adverse outcomes (15% *vs* 14%, 1·45, 0·73–2·90) or gestation at delivery (36·6 weeks *vs* 36·8 weeks; mean difference −0·52, 95% CI −0·63 to 0·73).

**Interpretation:**

We found that the availability of PlGF test results substantially reduced the time to clinical confirmation of pre-eclampsia. Where PlGF was implemented, we found a lower incidence of maternal adverse outcomes, consistent with adoption of targeted, enhanced surveillance, as recommended in the clinical management algorithm for clinicians. Adoption of PlGF testing in women with suspected pre-eclampsia is supported by the results of this study.

**Funding:**

National Institute for Health Research.

**Research in context**

**Evidence before this study**

We searched PubMed, ClinicalTrials.gov, and the ISRCTN registry for original articles published in English before Oct 1, 2018, with the search terms “pre-eclampsia AND angiogenic factor OR placental growth factor AND/OR trial”. We found no published trials that evaluated the implementation of angiogenic factors (such as placental growth factor [PlGF]) as a diagnostic adjunct in women presenting with suspected pre-eclampsia. However, we identified three trials from registry searches. We found a small (n=366), single-centre, retrospectively registered clinical trial, but the results of this trial have not yet been published. Two other multicentre trials are ongoing (still recruiting) in Ireland and Spain, both of which aim to evaluate whether incorporation of PlGF-based testing into current management algorithms improves maternal and perinatal outcomes in women with suspected pre-eclampsia. Previous cohort studies have demonstrated high sensitivity and a negative predictive value of PlGF-based testing in determining the need for delivery in women with suspected pre-eclampsia, but this testing has not been assessed within a trial.

**Added value of this study**

Our stepped-wedge cluster-randomised controlled trial provides evidence of the clinical effects of PlGF-based testing on the time to diagnosis and maternal and perinatal outcomes in women with suspected pre-eclampsia. In 11 hospitals across the UK, we implemented PlGF-based testing alongside a clinical management algorithm that was based on national guidance. We found a significant reduction in the time taken for a clinician to make a diagnosis of pre-eclampsia when using PlGF-based testing relative to routine clinical care. This approach was found to be associated with a reduction in severe maternal adverse outcomes, with no difference seen in gestational age at delivery or perinatal adverse outcomes. To our knowledge, our study is the first multicentre randomised controlled trial of PlGF-based testing as a diagnostic adjunct for women with suspected pre-eclampsia.

**Implications of all the available evidence**

Angiogenic factors have previously shown good performance in determining the need for delivery in women presenting with suspected pre-eclampsia. Our trial has demonstrated that, with implementation of PlGF-based testing, clinicians make a quicker diagnosis of pre-eclampsia and there is an associated reduction in serious maternal adverse events. The clinical use of PlGF measurement could present a change for antenatal care that improves speed of diagnosis and improves outcomes in pregnancy. Our findings provide novel evidence supporting the adoption of PlGF testing as a diagnostic adjunct for suspected pre-eclampsia. Evaluation of the intervention with women stratified by PlGF category could further elucidate the mechanisms by which PlGF testing and our management algorithm affect maternal outcomes.

## Introduction

Hypertension affects 10% of pregnant women, and pre-eclampsia complicates around 3% of singleton pregnancies.^1^, ^2^ Women with pre-eclampsia are often asymptomatic, even those with severe disease. Diagnosis is based on clinical features such as hypertension and raised urinary protein excretion, both of which are subject to observer error,^3^ heterogeneity in test accuracy,^4^, ^5^ and an insufficient ability of clinicians to predict important adverse pregnancy outcomes.^6^ The presentation of pre-eclampsia is often clinically ambiguous, and risk stratification of women with suspected pre-eclampsia is complex. This ambiguity leads to repeated hospital attendances for antenatal monitoring, increased use of health resources,^7^ and considerable anxiety for women, while missing at-risk cases.^8^

Angiogenic factors are associated with the pathophysiology of pre-eclampsia.^9^, ^10^ In a study^11^ of the accuracy of tests in diagnosing pre-eclampsia, low circulating maternal placental growth factor (PlGF) concentrations had a high sensitivity (96%; 95% CI 89–99) and negative predictive value (98%; 93–99·5) in diagnosing pre-eclampsia that required delivery within 14 days in women who presented with suspected pre-eclampsia. In this study, the area under the receiver operating characteristic curve for low PlGF concentrations in determining pre-eclampsia was 0·87 (SE 0·03), which is greater than all commonly used tests in the maternity assessment setting (such as for blood pressure, alanine transaminase, and urate).

Many diagnostic tests enter clinical care without being evaluated in a trial to assess whether the test makes a difference to diagnosing the condition it is intended for, whether use of the test affects downstream outcomes when implemented into practice, or whether diagnostic test performance is maintained in a real-world setting. There is a need to determine whether measurement of these novel angiogenic factors (such as PlGF) in pregnant women could translate into improved diagnosis and care when implemented into clinical practice. We therefore aimed to determine whether knowledge of circulating PlGF concentration, integrated with a clinical management algorithm, decreased the time for clinicians to make a diagnosis in women with suspected pre-eclampsia. We also aimed to determine whether this approach reduced subsequent maternal or perinatal adverse outcomes.

## Methods

### Study design and participants

We did a multicentre, pragmatic, stepped-wedge cluster-randomised controlled trial in 11 UK maternity units, which were each responsible for 3000–9000 deliveries per year. Women aged 18 years and older who presented with suspected pre-eclampsia between 20 weeks and 0 days and 36 weeks and 6 days of gestation, with a live, singleton fetus were invited to participate. Women were identified as having suspected pre-eclampsia at routine antenatal appointments or following an acute clinical presentation with symptoms, but women with a documented diagnosis of pre-eclampsia at presentation were ineligible. Suspected pre-eclampsia was defined as new-onset or worsening of existing hypertension, dipstick proteinuria, epigastric or right upper-quadrant pain, a headache with visual disturbances, fetal growth restriction, or abnormal maternal blood tests that were suggestive of disease (such as thrombocytopenia or hepatic or renal dysfunction). Since a very low PlGF result might have been concerning to some health-care professionals and women, with the unintended effect of increasing non-indicated preterm delivery, we made it clear in written information and training that a PlGF result was not an indication for delivery in itself. All participants provided individual written consent. The trial was approved by the London South East Research Ethics Committee (no. 15/LO/2058).

### Randomisation and masking

Women were approached individually, they consented to inclusion in the study, and they were asked for blood samples. We randomly allocated the maternity units, representing the clusters, to blocks. Blocks represented an intervention initiation time, which occurred at equally spaced 6-week intervals throughout the trial. At the start of the trial, all units had usual care (in which PlGF measurements were also taken but were concealed from clinicians and women). At the initiation time of each successive block, a site began to use the intervention (in which the circulating PlGF measurement was revealed and a clinical management algorithm was used). Enrolment of women continued for the duration of the blocks either to concealed PlGF testing (before implementation) or, after implementation, to revealed PlGF testing. The centres were randomly allocated to the order in which the intervention was introduced by the trial statistician (PTS), who did the trial analyses. This random allocation accounted for the number of deliveries per month, such that the rank correlation between delivery rate and the order of randomisation was zero. We used the rank correlation to balance the allocation order by number of women delivering per month, so the sites were evenly distributed between blocks in this regard, ensuring that the larger units or smaller units did not all receive the intervention at the beginning or the end of the trial. PlGF measurement was not routinely available in any of the sites before the trial and, for the duration of the trial, PlGF measurement was not used outside of the study in any of the sites, as confirmed on site visits and team teleconferences throughout the trial.

### Procedures

Before implementation of the intervention, in the usual care time period, additional blood samples were taken from all women participating and were processed at each unit within 4 h of sampling on an electronically-masked Triage instrument (Quidel Cardiovascular Inc; San Diego, CA), according to the manufacturer's instructions, which confirmed that the test had been successfully processed but without revealing a result. The usual care pathway followed local hospital practice, the National Institute for Health and Care Excellence (NICE) guidelines for management of hypertension in pregnancy,^12^ and national guidance for management of fetuses suspected to be small for gestational age.^13^

The trial consisted of 12 6-week blocks. One unit transitioned to the intervention at the start of each block, following an initial block in which all maternity units used usual care with concealed testing. We informed units 6 weeks before their transition to the intervention, to allow time for training in the incorporation of PlGF measurements into current NICE management of hypertension in pregnancy, the mainstay of management. At transition, a meter to show revealed results and a training package, including a management algorithm, were provided; this package incorporated PlGF measurement into the NICE guidance for the management of hypertensive pregnancies (appendix). A short training session was given by the trial team to describe integration of PlGF testing into current management guidelines. At the time of the last block, all participating units were using this intervention.

### Outcomes

All outcomes were assessed on an individual level by intention to treat. The primary outcome was the time in days from trial entry to a documented diagnosis of pre-eclampsia, as defined by the International Society for the Study of Hypertension in Pregnancy (ISSHP) 2014 statement,^14^ in each woman's clinical notes. PlGF measurements were not a component of the clinical diagnosis, since this test is not included within the ISSHP diagnostic criteria. Every diagnosis, including all diagnosed cases of pre-eclampsia, was also reviewed by a central adjudication panel who were masked to trial allocation. All diagnosed cases of pre-eclampsia were verified by a central adjudication team consisting of a clinical doctor and a research midwife, who were also masked to site allocation and PlGF result, at periodic data review meetings throughout the trial. The time to diagnosis was reviewed in the case notes, and all cases had to meet the ISSHP criteria for diagnosis of pre-eclampsia. Any discrepancies between the notes and the adjudication panel were sent for independent review by the chief investigator, who remained masked to group allocations until data were analysed.

The prespecified secondary maternal outcomes were a composite of severe maternal adverse events, as defined by the fullPIERS consensus.^15^ These adverse outcomes were reported as the number of women with one event or more of: maternal death, eclampsia, a Glasgow Coma Scale score of less than 13, stroke, transient ischaemic attack, cortical blindness or retinal detachment, posterior reversible encephalopathy, a requirement for positive inotropic support, a requirement for parenteral infusion of a third-line antihypertensive, myocardial ischaemia or infarction, blood oxygen saturations of less than 90%, 50% FiO_2_ (or higher) for more than 1 h, a requirement for intubation (other than for caesarean section), pulmonary oedema, a requirement for transfusion of blood products, a platelet count of less than 50 × 10^9^ platelets per L, hepatic dysfunction, haematoma or hepatic rupture, severe acute kidney injury (defined as concentrations of creatinine >150 μmol/L or >200 μmol/L in chronic kidney disease, a requirement for dialysis), or placental abruption. We also tested for the secondary maternal outcomes of systolic blood pressure of more than 160 mm Hg, progression to severe pre-eclampsia, placental abruption, mode and onset of delivery, and PlGF test performance in women presenting before 35 weeks of gestation and that in women presenting between 35 weeks and 0 days and 36 weeks and 6 days of gestation for clinically-indicated delivery within 14 days of trial entry. Because of concerns over selection bias, we have reported and tested the proportion of women reaching the diagnostic criteria, irrespective of clinical confirmation of pre-eclampsia. We also assessed several additional descriptive secondary maternal outcomes post hoc, after peer review.

The prespecified secondary perinatal outcomes were gestation at delivery, preterm birth before 37 weeks of gestation, birthweight and birthweight centile,^16^ Apgar scores at 5 min after birth, admission to a neonatal unit, perinatal death (defined as deaths from 24 weeks of gestation, including those defined as stillbirths, until 7 completed days after birth), and late neonatal deaths (deaths between 8 and 27 complete days after birth). Severe perinatal morbidity and mortality was an additional secondary composite perinatal outcome that was assessed post hoc, which comprised the number of babies with one event or more of: necrotising enterocolitis stage 2 and 3, retinopathy of prematurity, intraventricular haemorrhage, respiratory distress syndrome, seizures, bronchopulmonary dysplasia, stillbirth, early neonatal death, and late neonatal death before or at 28 days.

Maternal health care resource use outcomes comprised outpatient attendances and inpatient nights in hospital. Neonatal resource use outcomes were nights in the highest-level care (intensive and high-dependency care) and special care.^17^ These outcomes were prespecified.

Two prespecified sensitivity analyses on the primary outcome were done: the first analysis was for all women with a pre-eclampsia diagnosis (both a clinical diagnosis, and a diagnosis following adjudication by the trial team), and the second analysis was for women diagnosed within 4 weeks of enrolment, censoring diagnoses at 4 weeks from trial entry.

### Statistical analysis

We based our estimates on the PELICAN study,^11^ from which we assumed that 58% of women presenting with suspected pre-eclampsia will later be diagnosed with the disease. We specified a high intracluster correlation (0·3) to confirm that we would have adequate power under plausible circumstances. We required at least 12 women with suspected pre-eclampsia per unit (in six maternity units) per timeframe, giving a required sample size of 504 women (from which we would find an estimated 294 cases). This design and sample size gave us more than 95% power (by the method by Hemming and colleagues)^18^ to show a 50% reduction in mean time to diagnosis from 14 days (SD 14) to 7 days, assuming a minimum of six maternity units. We chose a 50% reduction for time to diagnosis as a possible effect of the intervention that was plausible, notable, and likely to affect clinical practice.

At the trial planning stage, 11 maternity units confirmed participation and, post hoc, our intracluster correlation was 0·035, which was much lower than we allowed for in the power calculation (thereby increasing the study power). However, the actual proportion of women diagnosed with pre-eclampsia was found to be lower than anticipated. This reduced proportion of women diagnosed was offset by more sites participating than originally planned.

The primary outcome (time to diagnosis with pre-eclampsia) was assessed by linear regression of logged time to diagnosis by use of robust standard errors^19^ (in those who were clinically diagnosed with pre-eclampsia) and was presented as adjusted ratios of geometric means. We adjusted for centre, time, gestational age at entry, and three covariates prespecified to potentially affect the primary outcome: previous pre-eclampsia, chronic hypertension, and chronic kidney disease. A log transformation was used because the data were log-normally distributed when data were inspected before unmasking. We did not include attending clinician in the model because pregnant women in the UK National Health Service health-care setting are routinely managed by several health-care professionals (including obstetricians and midwives), with clinical decisions often being made by a consensus among multiple clinicians. The relevant dates and times were entered on the database with a 24-h clock, but time to diagnosis was presented in days (rather than hours) because this approach is the most pragmatic (when time progresses beyond 24 h) and clinically meaningful. One prespecified sensitivity analysis was done with linear regression of all women with a pre-eclampsia diagnosis by either the clinicians or the trial team, with undiagnosed pre-eclampsia treated as diagnosed at delivery. For the other sensitivity analysis, we used a parametric survival analysis on data from all women with a pre-eclampsia diagnosis, censoring diagnoses at 4 weeks from trial entry. We prespecified the 4-weeks timepoint to reflect previous studies^20^ that have reported time to delivery within 4 weeks.

Secondary outcomes were analysed with linear regression, adapting with log transformation where appropriate. All binary outcomes were analysed with a binomial regression model with a log link. Test performance was evaluated with sensitivity, specificity, positive and negative predictive values, and positive and negative likelihood ratios. Mixed-effects log-normal regression curves were generated for the proportion of women diagnosed relative to time from trial entry. Health-care resource use was analysed with generalised linear mixed models with linear time-fixed effects and random effects for centre. We chose the best performing model (using suitable family and link functions) based on the lowest Akaike Information Criteria.

To account for the stepped-wedge design and potential for selection bias, differences in prespecified, clinically relevant baseline characteristics were tested with logistic regression for binary variables and ordered logistic regression for ordered categorical variables. If there were more than 5% of participants with missing data for the primary outcome, further adjustment would be considered. All outcomes were adjusted for centre and categorical time effects because of the trial design. Effects were estimated with multiple regression, including terms for the intervention with fixed effects using dummy variables at each time in each centre. Centre was considered as a categorical variable and fitted as separate dummy variables for each centre. Calendar time was treated as a single categorical time variable.

On central review, some women were identified as having fulfilled the ISSHP criteria for pre-eclampsia but were never given this diagnosis by the clinical team, and hence were missed. These women were recorded as having pre-eclampsia by adjudication but were not included in the prespecified primary outcome analysis because they did not have a clinical diagnosis of pre-eclampsia. Results are reported as per CONSORT guidelines.^21^ All statistical analyses were done with Stata version 14.2 (StataCorp; College Station, TX). This trial is registered with ISRCTN, number 16842031.

### Role of the funding source

The funder of the study had no role in study design, data collection, data analysis, data interpretation, or writing of the report. The corresponding author had full access to all the data in the study and had final responsibility for the decision to submit for publication.

## Results

**Figure 1:**
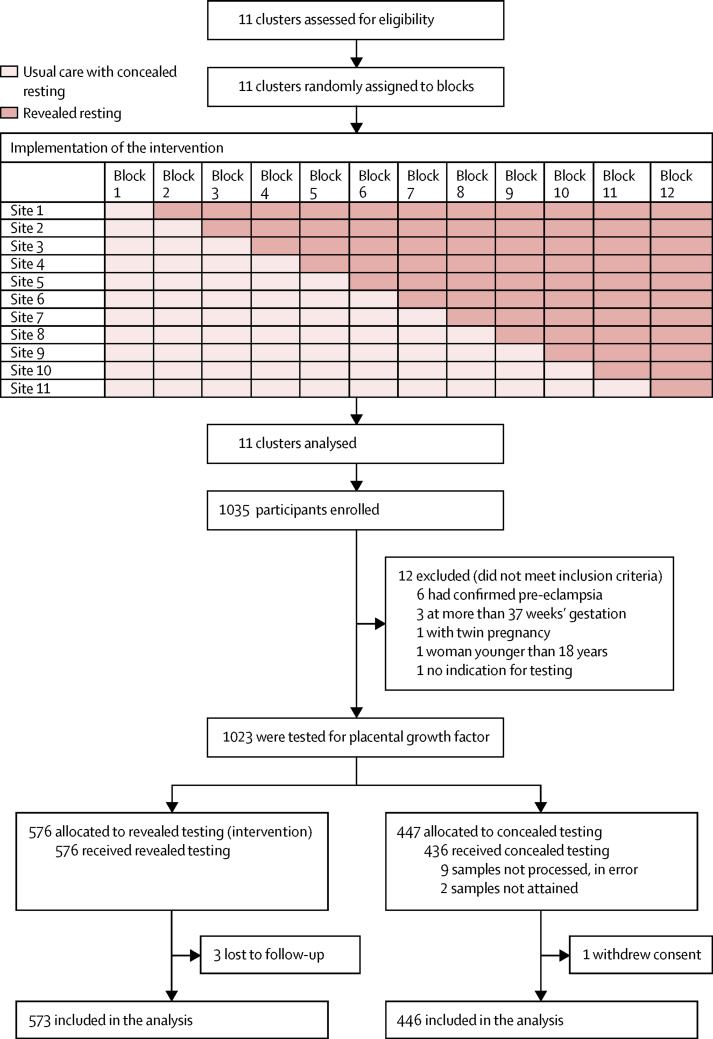
Trial profile

Between June 13, 2016, and Oct 27, 2017, 1035 women at 11 maternity units were enrolled and assessed for their inclusion in the trial; 12 (1%) women were found to be ineligible (figure 1). Of the 1023 eligible women, 576 (56%) women were included in the trial while their maternity unit was allocated to the intervention (revealed testing) group, and 447 (44%) women were included in the trial while their maternity unit was allocated to the concealed testing group. All women allocated to the revealed testing group received this intervention, but only 436 (98%) women allocated to the concealed testing group received this treatment because samples from nine women at one site were not processed due to an administrative error, and a sample was not obtained from two women at other sites. Three (1%) women in the revealed testing group were lost to follow-up, so 573 (99%) women in this group were included in the analyses. One (<1%) woman in the concealed testing group withdrew consent to follow-up data collection, so 446 (>99%) women in this group were included in the analyses. No further analyses were needed to account for missing data (in <1% of women).

There was no contamination between the trial groups—ie, no National Health Service identification numbers were duplicated in the database. The trial team kept strict logs of supply and use of the Triage PlGF cartridges, and a consent form was required to use each kit. There were no unauthorised tests on the data downloads from the Triage meters. The number of women recruited by trial centre is shown in the appendix. Participant characteristics are shown in table 1. The two groups were comparable at trial entry, and no substantial differences were observed in baseline variables.

**Table 1:** Baseline demographics and clinical characteristics

		**Revealed PlGF (intervention; n=576)**	**Concealed PlGF (n=447)**
Age, years		31·9 (5·9)	31·5 (6·0)
Ethnicity			
	White	378 (66%)	292 (65%)
	Black	76 (13%)	63 (14%)
	Indian, Pakistani, Bangladeshi, or Sri Lankan	67 (12%)	52 (12%)
	Mixed	13 (2%)	11 (2%)
	Other (including Chinese)	39 (7%)	26 (6%)
Body-mass index, kg/m^2^		27·9 (23·9–33·1)	28·4 (24·2–34·1)
Previous pregnancies with durations of 24 weeks or more			
	0	317 (55%)	211 (47%)
	1	133 (23%)	120 (27%)
	2	59 (10%)	65 (15%)
	≥3	67 (12%)	51 (11%)
Number of previous pregnancies with durations of less than 24 weeks*			
	0	449 (78%)	318 (71%)
	1	65 (11%)	81 (18%)
	≥2	45 (8%)	47 (11%)
Previous pre-eclampsia (of multiparous women)		99 (38%) of 259	92 (39%) of 236
Pre-existing chronic hypertension		87 (15%)	70 (16%)
Pre-existing renal disease		24 (4%)	17 (4%)
Diabetes before pregnancy		31 (5%)	26 (6%)
Blood pressure at initial antenatal visit, mm Hg			
	Systolic	120 (15)	120 (16)
	Diastolic	74 (11)	72 (12)
Being prescribed prophylactic aspirin		235 (41%)	178 (40%)
Has gestational diabetes		71 (12%)	53 (12%)
Presenting signs and symptoms†			
	New-onset hypertension	299 (52%)	209 (47%)
	Worsening of existing hypertension	100 (17%)	79 (18%)
	New-onset proteinuria	341 (59%)	263 (59%)
	Epigastric or right upper-quadrant pain	47 (8%)	47 (11%)
	Neurological symptoms	187 (32%)	150 (34%)
	Suspected fetal growth restriction	103 (18%)	62 (14%)
	Abnormal blood test results	19 (3%)	8 (2%)
	Reduced fetal movement	6 (1%)	5 (1%)
Gestation at enrolment, weeks		32·3 (3·8)	32·7 (3·9)
Highest blood pressure in the 48 h before study entry, mm Hg			
	Systolic	144 (20)	143 (20)
	Diastolic	91 (14)	91 (13)
Highest dipstick proteinuria in the 48 h before study entry			
	None	207 (37%)	170 (38%)
	Trace	60 (11%)	57 (13%)
	1+	153 (27%)	108 (24%)
	≥2+	147 (26%)	111 (25%)
Mean PlGF, pg/mL		186 (277)	202 (355)
Median PlGF, pg/mL		55 (13–235)	39 (12–236)
PlGF, pg/mL			
	<12	130 (23%)	106 (24%)
	12–100	213 (37%)	173 (39%)
	>100	230 (40%)	167 (37%)

Data are mean (SD), n (%), or median (IQR). PlGF=placental growth factor.

* Including previous miscarriages, ectopic pregnancies, and terminations of pregnancy at less than 24 weeks of gestation.

† Women could have more than one sign or symptom.

In the concealed testing group, low PlGF (<100 pg/mL) concentrations had a high accuracy in determining pre-eclampsia that required delivery within 14 days; this test had high sensitivity (94·9%) and negative predictive values (98·3%) in women presenting before 35 weeks of gestation (appendix). In women presenting between 35 weeks and 0 days and 36 weeks and 6 days of gestation, the sensitivity was 96·2% and the negative predictive value for delivery before 37 weeks was 97·1%.

**Figure 2:**
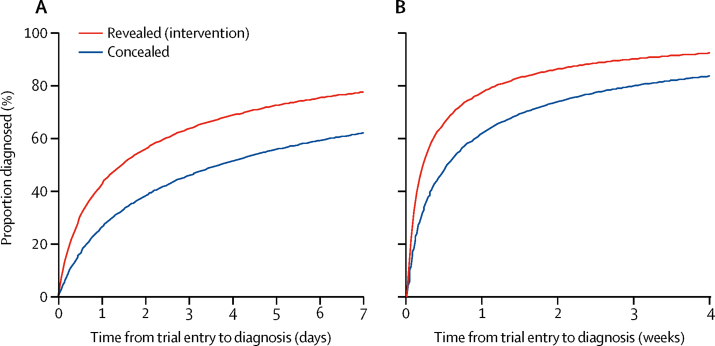
Proportion of women diagnosed with pre-eclampsia when revealing versus concealing circulating placental growth factor concentrations from clinicians, over days (A) and weeks (B) Data are mixed-effects log-normal regression curves.

The median time to diagnosis of pre-eclampsia was 1·9 days in the revealed testing group versus 4·1 days in the concealed testing group. The time ratio was 0·36 (95% CI 0·15–0·87; p=0·027), corresponding to a 64% reduction in time to diagnosis (13–85%; table 2). The intra-cluster correlation for the primary outcome was 0·035. 205 (36%) women in the revealed testing group and 155 (35%) women in the concealed testing group were diagnosed with pre-eclampsia. The mixed-effects log-normal regression curves of the proportion of women diagnosed by time from trial entry with revealed versus concealed PlGF testing are shown in figure 2. More women were diagnosed within 24 h of enrolment in the revealed testing group (52 [20%] women) compared with those in the concealed testing group (31 [16%] women; adjusted odds ratio [aOR] 3·6, 95% CI 1·16–11·20; p=0·027). The incidence of severe pre-eclampsia did not differ between the groups (1·22 [0·71–2·12])

**Table 2:** Primary and secondary maternal outcomes

		**Revealed PlGF (intervention; n=573)**	**Concealed PlGF (n=446)**
**Primary outcome**			
Number diagnosed with pre-eclampsia		205 (36%)	155 (35%)
Time to diagnosis of pre-eclampsia in those diagnosed, days		1·9 (0·5–9·2)	4·1 (0·8–14·7)
**Secondary maternal outcomes**			
Number of women with adverse outcomes, defined by the fullPIERS consensus*		22 (4%)	24 (5%)
	Maternal deaths	0	0
	Eclampsia	0	2 (<1%)
	Stroke	0	2 (<1%)
	Parenteral infusion of third-line antihypertensive required	1 (<1%)	3 (1%)
	Myocardial infarction	0	1 (<1%)
	Blood oxygen saturation <90%	1 (<1%)	1 (<1%)
	Intubation required (other than for caesarean section)	0	1 (<1%)
	Pulmonary oedema	2 (<1%)	0
	Transfusion of blood products required	9 (2%)	14 (3%)
	Platelet count <50 × 10^9^ platelets per L	4 (1%)	4 (1%)
	Hepatic dysfunction	1 (<1%)	0
	Severe acute kidney injury	7 (1%)	6 (1%)
	Dialysis required	0	1 (<1%)
	Placental abruption	4 (1%)	5 (1%)
Primary diagnosis			
	Pre-eclampsia	175 (31%)	126 (28%)
	Superimposed pre-eclampsia	30 (5%)	29 (7%)
	Gestational hypertension	100 (17%)	77 (17%)
	Gestational proteinuria	29 (5%)	20 (4%)
	Small-for-gestational-age infant only	32 (6%)	28 (6%)
	Chronic hypertension only	37 (6%)	28 (6%)
	Chronic hypertension with a small-for-gestational-age infant	11 (2%)	9 (2%)
	Renal disease	7 (1%)	4 (1%)
	Transient hypertension	8 (1%)	20 (4%)
	None of the above	94 (16%)	63 (14%)
Subsequent diagnosis of pre-eclampsia by adjudication team		50 (9%)	42 (9%)
Number with pre-eclampsia, diagnosed by adjudication		255 (44%)	197 (44%)
Severe pre-eclampsia		155 (27%)	106 (24%)
Time to diagnosis of pre-eclampsia (of those diagnosed within 4 weeks of trial entry), days		1·3 (0·3–6·0)	2·7 (0·7–8·9)
Fetal growth abnormalities on ultrasound*			
	Received a scan	438 (77%)	307 (69%)
	Any growth abnormality identified	142 (25%)	67 (22%)
	Estimated fetal weight of less than the tenth percentile	117 (27%)	62 (20%)
	Absent or reversed end-diastolic flow	43 (10%)	16 (5%)
Use of antihypertensives		347 (61%)	270 (61%)
Systolic blood pressure of at least 160 mm Hg		239 (42%)	188 (42%)
Labour onset			
	Spontaneous	79 (14%)	78 (17%)
	Induced	263 (46%)	210 (47%)
	Pre-labour caesarean section	230 (40%)	158 (35%)
Preterm deliveries <37 weeks		234 (41%)	167 (37%)
Indication for induction or caesarean section before labour†			
	Maternal hypertension not controlled by maximal therapy	25 (5%)	28 (8%)
	Maternal haematological abnormality	10 (2%)	3 (1%)
	Maternal biochemical abnormality	15 (3%)	16 (4%)
	Fetal compromise on ultrasound	34 (7%)	19 (5%)
	Fetal compromise on cardiotocography	31 (6%)	40 (11%)
	Severe maternal symptoms of pre-eclampsia	48 (10%)	27 (7%)
	Diagnosis of pre-eclampsia and reaching 37 weeks of gestation	65 (13%)	57 (16%)
	Gestational hypertension and reaching 37 weeks of gestation	56 (11%)	37 (10%)
	Chronic hypertension and reaching 37 weeks of gestation	27 (6%)	17 (5%)
	Enrolled in PHOENIX trial	13 (3%)	9 (2%)
	Other obstetric complications	170 (34%)	115 (31%)
Mode of delivery			
	Spontaneous vaginal cephalic	210 (37%)	182 (41%)
	Assisted vaginal (forceps or vacuum)	42 (7%)	38 (9%)
	Vaginal breech	1 (<1%)	2 (<1%)
	Pre-labour caesarean section	170 (30%)	130 (29%)
	In-labour caesarean section	150 (26%)	94 (21%)
Major post-partum haemorrhage		49 (9%)	48 (11%)
Maternal health resource use			
	Mean outpatient visits (SE)	6·14 (0·53)	9·44 (0·81)
	Mean inpatient nights (SE)	7·43 (0·36)	7·26 (0·38)

Data are n (%), median (IQR), or mean (SD), unless otherwise indicated. For all fullPIERS outcome data not provided, no women had any of these events. Hepatic dysfunction was defined as an international normalisation ratio of more than 1·2 in the absence of disseminated intravascular coagulation (defined as abnormal bleeding and consumptive coagulopathy—ie, low platelets, abnormal peripheral blood film, or any of increased international normalisation ratio, increased activated partial thromboplastin time, low fibrinogen, or increased fibrin degradation products that are outside normal non-pregnancy ranges) or treatment with warfarin. Time ratio for the time to diagnosis of pre-eclampsia (primary outcome) 0·36 (95% CI 0·15–0·87). PlGF=placental growth factor.

* Women could have several adverse events.

† Of 494 women in the revealed group and 368 women in the concealed group.

Severe maternal adverse outcomes^22^ were less frequent in the revealed testing group than the concealed testing group (22 [4%] *vs* 24 [5%] events; aOR 0·32, 95% CI 0·11 to 0·96; p=0·043). There were five serious events (two eclamptic fits, two strokes, and one cardiac arrest in four women, all of whom had low PlGF concentrations) in the concealed testing group, whereas there were no similarly serious events in the revealed testing group (table 2). A higher proportion of women in the concealed testing group than the revealed testing group received transfusion of blood products (14 [3%] *vs* 9 [2%]). The highest mean systolic blood pressure, use of antihypertensive medication, and use of magnesium sulphate did not differ between groups. More women in the revealed testing group than the concealed testing group received a fetal ultrasound (438 [77%] *vs* 307 [69%] women) and a higher umbilical artery pulsatility index was seen in the revealed testing group (more than the 95th percentile in 66 [16%] *vs* 27 [9%] women; 2·94, 1·07 to 8·11). The number of fetuses with a birthweight of less than the tenth centile (1·49, 0·70 to 3·15) and those with absent or reversed end diastolic flow rates (1·82, 0·56 to 5·90) did not differ between the groups. Both groups showed a similar frequency of pre-labour caesarean sections. Delivery gestation did not differ significantly between the groups (36·6 weeks in the revealed testing group *vs* 36·8 weeks in the concealed testing group; mean difference −0·52, 95% CI −0·63 to 0·73).

The composite adverse perinatal outcome did not differ between the groups: 85 (15%) fetuses in the revealed testing group and 63 (14%) fetuses in the concealed testing group had adverse events (aOR 1·45, 95% CI 0·73–2·90; table 3). We observed no differences in the prevalence of spontaneous vaginal delivery (1·05, 0·59–1·86), or the use of pre-labour caesarean sections (0·96, 0·59–1·55) or in-labour caesarean sections (0·78, 0·48–1·25) between the groups. We also observed no differences in perinatal deaths, preterm delivery before 37 weeks of gestation (1·00, 0·61–1·63), birthweight centiles, neonatal unit admissions, gestation at delivery, or indication for delivery with PlGF testing (appendix).

**Table 3:** Secondary perinatal outcomes

		**Revealed PlGF (intervention; n=573)**	**Concealed PlGF (n=446)**
Gestation at delivery, weeks		36·6 (3·0)	36·8 (3·0)
Vital status at birth			
	Livebirth	567 (99%)	440 (99%)
	Intrauterine fetal death	7 (1%)	6 (1%)
Perinatal adverse outcomes*		86 (15%)	63 (14%)
Composite perinatal adverse outcome components			
	Any grade of intraventricular haemorrhage	7 (1%)	11 (3%)
	Seizure	0	2 (<1%)
	Any grade of retinopathy of prematurity	9 (2%)	9 (2%)
	Respiratory distress syndrome	78 (14%)	54 (12%)
	Bronchopulmonary dysplasia	5 (1%)	3 (1%)
	Necrotising enterocolitis (stage 2 or 3)	7 (1%)	7 (2%)
Perinatal deaths		6 (1%)	4 (1%)
Late neonatal deaths (8–27 complete days of life)		3 (1%)	1 (<1%)
Neonatal unit admission		195 (34%)	146 (33%)
Birthweight, g		2657 (887)	2720 (858)
Birthweight centile		42·8 (33·0)	43·4 (33·1)
Apgar score at 5 min after delivery		9·14 (1·52)	9·24 (1·41)
Umbilical arterial pH at birth		7·25 (0·11)	7·23 (0·09)
Umbilical arterial pH <7·2 at birth		141 (25%)	135 (30%)
Perinatal health resource use (in those admitted)			
	Inpatient nights in the neonatal unit	22·1 (25·9)	24·6 (35·2)
	Nights in the intensive care or high-dependency units	15·2 (1·7)	24·2 (3·8)
	Nights in the special care baby unit	14·7 (14·4)	13·09 (12·6)

Data are mean (SD), n (%), or median (IQR). Fetuses could have one or several composite perinatal adverse components. PlGF=placental growth factor.

* Only components with an outcome are included here; full details are shown in the appendix.

Prespecified serious adverse events are shown in table 4. No maternal serious adverse events were reported in the revealed testing group versus five events in four (1%) women in the concealed testing group. There were similar numbers of fetal and neonatal deaths in both groups (ten deaths in the revealed testing group versus seven deaths in the concealed testing group). We found that a low circulating PlGF concentration preceded all morphologically normal fetuses that were stillborn except one (table 4).

**Table 4:** Prespecified serious adverse events

		**Revealed PlGF (intervention; n=573)**	**Concealed PlGF (n=446)**
Number of women with maternal adverse events		0	4 (1%)
	Maternal death	0	0
	Maternal stroke	0	2 (<1%)
	Maternal cardiac arrest	0	1 (<1%)*
	Eclampsia	0	2 (<1%)
Number of babies with perinatal serious adverse events		10 (2%)	7 (2%)
Intrauterine fetal death			
	Before viability†	3 (1%); 2	3 (1%); 3
	Viable (no fetal dysmorphism)	1 (<1%); 1	3 (1%); 2‡
	Viable (fetal dysmorphism noted)	3 (1%); 1	0
Neonatal deaths		3 (1%); 3	1 (<1%); 1

Data are n (%); for fetal events, the number of mothers with a PlGF concentration of less than the fifth centile is presented after the semicolon. PlGF=placental growth factor.

* This cardiac arrest occurred as an additional event to a stroke.

† Less than 24 weeks of gestation or less than 500 g.

‡ One sample was not processed.

A similar proportion of women met the diagnostic criteria for pre-eclampsia after adjudication in both groups (255 [44%] women in the revealed testing group and 197 [44%] women in the concealed testing group). In women diagnosed with pre-eclampsia by adjudication, diagnosis was reached by the treating clinicians in 205 (80%) women in the revealed testing group compared with 155 (79%) women in the concealed testing group.

The revealed testing group had a mean of 6·14 (SE 0·53) antenatal outpatient attendances versus 9·44 (0·81) visits in the concealed testing group; however, this difference was removed after negative binomial regression adjustment for calendar time (negative binomial −0·04, 95% CI −0·23 to 0·16). Women in the revealed testing group spent a mean of 7·43 (SE 0·36) nights in inpatient care, versus 7·26 (0·38) nights in the concealed testing group (−0·06, −0·22 to 0·09). Women in the revealed testing group used the highest levels of neonatal care (intensive and high-dependency care nights) to a lesser extent (a mean of 15·2 [SE 1·7] nights *vs* 24·2 [3·8] nights in the concealed testing group (mean difference −10·6, 95% CI −20·81 to −0·47), and we found no difference between the groups in the number of nights spent in the special care baby unit.

## Discussion

Our trial has shown that, in women presenting with suspected pre-eclampsia, PlGF measurement, incorporated into a management algorithm based on national guidelines, significantly reduces the time taken for treating clinicians to diagnose pre-eclampsia. This improvement was associated with a significant reduction in maternal adverse outcomes, with no detected difference in gestational age at delivery or adverse perinatal outcomes. Revealing PlGF results did not change the clinical indications for elective early delivery within our trial population. Additionally, we found no increase in neonatal unit admissions, but we noted a reduction in the use of higher levels of neonatal care when revealing PlGF results. For more than 100 years, pre-eclampsia diagnosis has relied on poorly reproducible clinical signs, such as blood pressure and proteinuria; PlGF measurement could present a change for antenatal care that improves not only diagnosis, but also management and pregnancy outcome.

To our knowledge, this is the first multicentre randomised controlled trial of PlGF measurement as a diagnostic adjunct for pre-eclampsia. Trials to assess the effects of such tests on clinical care are uncommon. The Patient Centred Outcomes Research Institute recommend that process of care outcomes (such as time to procurement of a definitive diagnosis) should be used to evaluate diagnostic tests alongside patient-centred outcomes such as morbidity or mortality outcomes.^23^ This recommendation reflects the need for trials of diagnostic tests to evaluate both the benefits of the intervention in a diagnostic capacity, and also the effects on downstream clinical outcomes. A reduction of around 2 days in clinicians reaching a diagnosis of preterm pre-eclampsia is important for decision making in a disease with a substantial degree of diagnostic uncertainty. Such a decision would usually affect the care pathway regarding the place and frequency of management (including whether to transfer the pregnant woman to a hospital with an appropriate neonatal care facility), avoidance of severe morbidity, administration of steroids for fetal lung maturity, and timing of delivery. The ability to diagnose pre-eclampsia more rapidly enables targeted, individualised management. There is a double benefit of appropriately reassuring women who do not need intensive investigation and minimising excessive health service use for those at lower risk within this acute period of diagnostic ambiguity.

We managed women in both trial groups in accordance with the national guidelines on the management of hypertension in pregnancy. However, the revealed PlGF group received the results of their PlGF measurement incorporated into this clinical guidance. This was a pragmatic trial, to reflect how angiogenic factor measurement could be adopted clinically and realistically within a health-care service. We provided simple guidance around the blood test result (as would be given in usual practice), with clinical management decisions of individual pregnant women left to the discretion of the treating clinician, including future assessments and schedules of care. The trial intervention was, of necessity, not masked to clinicians, and we hypothesised that the intervention might affect diagnostic decisions and management. We have aimed to report not only the test accuracy of PlGF, but its effect on the process and health outcomes of the pregnant women,^23^ mediated through clinician behaviour.

The trial was intentionally designed to evaluate real-world effectiveness of the intervention^24^ rather than efficacy in ideal conditions by mimicking adoption of the diagnostic test into clinical practice across several sites. Our trial included an assessment of process, clinical outcomes, and health resource use to evaluate the effects of the intervention across these domains.

The strengths of our study include that it comprised a robust evaluation of a test on both real-world diagnostic performance and clinical effects (beyond demonstrating test accuracy), the generalisability of the findings through testing across several sites, and inclusion of an ethnically and sociodemographically diverse population. The broad inclusion criteria for testing relate to usual antenatal presentations to obstetric triage. The provision of the management algorithm and short training package, with individual patient management left to the discretion of the treating clinician, reflect how PlGF testing might be used if adopted more widely. We anticipate that these findings would be generalisable to similar settings in which pregnant women present to physicians with suspected pre-eclampsia for assessment. All diagnoses were reviewed by the trial team, who were masked to trial allocation, with stringent diagnostic criteria. The analysis followed a prespecified analysis plan. Our trial also had some limitations: it was restricted to women with singleton pregnancies before 37 weeks of gestation and the findings might not be generalisable to pregnancies with multiple fetuses and those presenting with late-onset pre-eclampsia after 37 weeks of gestation, although women with gestational hypertension or confirmed pre-eclampsia presenting after 37 weeks of gestation are usually managed with planned delivery rather than surveillance.^25^ Because the greatest challenges associated with diagnostic uncertainty relate to women presenting before 37 weeks of gestation, in whom maternal and perinatal morbidity associated with pre-eclampsia is greatest, our trial has addressed an important time period in clinical management of pre-eclampsia. We chose a stepped-wedge design because we were aiming to introduce a biochemical test and associated management algorithm, necessitating change in practice across a service. Women and investigators were clear that this was their preferred study design because individual randomisation was perceived to be unfeasible, inequitable, and prone to contamination. However, this trial type might have confounding effects because of secular trends in calendar time, related to sequential implementation of the intervention across participating units, a potential limitation of such a design. We found a difference in recruitment numbers between the revealed and concealed testing groups due to higher recruitment numbers in later blocks (when more sites had transitioned to revealed testing). However, we did not restrict recruitment in the later stages of the trial to avoid selection bias. It is possible that the increase in numbers recruited in the intervention group could be a form of selection bias in itself, with clinicians recruiting different women due to the test becoming available, or that revealing the test result could affect the diagnosis of pre-eclampsia itself; these biases do not appear to be substantial because the baseline characteristics and the proportion diagnosed with pre-eclampsia were similar across both groups. Future stepped-wedge trials could consider a run-in phase, to mitigate against lower recruitment in the early months relative to later months.

Previous studies^11^, ^20^, ^26^, ^27^ have used a cohort design to investigate the potential usefulness of angiogenic factors for diagnosis of pre-eclampsia in women presenting with suspected pre-eclampsia, rather than randomised trials. We found similar test performance statistics in our concealed testing group to those seen in other large cohort studies^11^, ^20^ and clinical evaluation studies.^26^, ^27^ We did not report test performance in the intervention group to avoid the test performance for diagnosis of pre-eclampsia being affected by knowledge of the test result. We reported an additional perinatal composite outcome similar to that used previously^26^ to enable evaluation of the effects of the intervention on perinatal outcomes. We used PlGF testing, rather than the soluble fms-like tyrosine kinase 1/PlGF ratio as used in other studies.^20^ Evidence has shown that the commercially available tests compare similarly in their prediction of need for delivery within 14 days;^28^ we anticipate that these results would be generalisable in similar settings that use alternative PlGF assays. Other studies have assessed the detection frequency of subsequent pre-eclampsia by use of a combination of maternal factors, including mean arterial pressure, uterine artery pulsatility index, serum PlGF, and serum soluble fms-like tyrosine kinase-1 in unselected populations of women at fixed timepoints (eg, 20 weeks and 36 weeks).^29^, ^30^ External validation and economic analysis of these algorithms are awaited.

Suspected pre-eclampsia is one of the most frequent presentations to antenatal services, and current methods to risk stratify women are inadequate for accurate prediction of adverse outcomes. Our trial was powered to show a reduction in the time to diagnosis of pre-eclampsia and has shown that clinical use of PlGF does facilitate earlier diagnosis. More timely management, particularly relating to targeted surveillance of women at increased risk, could contribute to a reduction in maternal adverse events. The composite of severe maternal adverse outcomes was a prespecified secondary outcome measure, but the reduction found in the revealed testing group could be a chance finding requiring circumspect interpretation.

Low PlGF concentrations have been shown to have high sensitivity and negative predictive values for intrauterine fetal death,^11^ as confirmed in our trial. For pregnancies complicated by fetal growth restriction with a viable fetus between 26 and 32 weeks of gestation, there is evidence from long-term neurodevelopmental follow-up that management should be directed by monitoring of the ductus venosus waveform and computerised cardiotocography.^31^ Earlier confirmation of placental dysfunction in suspected fetal growth restriction by use of PlGF has the potential to improve risk stratification and target surveillance. However, given the low numbers of stillbirths in our study, further work is required to investigate the usefulness of PlGF for the prevention of later stillbirths.

In the revealed testing group, we found a lower duration of intensive and high-dependency care received by infants relative to the concealed testing group. Maternal health-care resource use did not differ between the groups. These findings might reflect more appropriate antenatal surveillance in at-risk pregnancies, including earlier recognition of fetal compromise and more timely delivery, with more appropriate stratification of those at lower risk.

Previous cohort studies^11^, ^20^ have suggested that test performance of angiogenic markers is accurate in ruling out pre-eclampsia. Further work is needed to clarify the potential effects on maternal and perinatal adverse outcomes as a primary powered endpoint, and studies are ongoing. Angiogenic markers vary throughout gestation, and there is a paucity of evidence regarding acute changes in angiogenic markers in pregnancies with persistent clinical disease suspicion. Our trial did not assess repeat PlGF testing in women with recurrent presentations who remained undiagnosed, and the optimum timing of repeat sampling remains uncertain. Furthermore, although our trial has evaluated the usefulness of PlGF with categories based on centile thresholds^11^ there could be additional benefit to the use of PlGF as a continuous measurement, in a similar manner to other clinically used biochemical variables. Finally, PlGF measurement could also be used in prognostic stratification after pre-eclampsia is confirmed as a diagnosis, particularly at gestations in which the balance between risks to mother and baby are difficult to judge.

In conclusion, the findings of our trial provide novel evidence supporting the adoption of PlGF testing as a diagnostic adjunct in women presenting with suspected pre-eclampsia.

## Data sharing

The trial dataset will be available to appropriate academic parties on request from the corresponding author, in accordance with the data sharing policies of King's College London, with input from the investigator group where applicable, subject to submission of a suitable study protocol and analysis plan, on publication of all initial trial results.
