# Supplementary material for: Placental growth factor testing to assess women with suspected pre-eclampsia: a multicentre, pragmatic, stepped-wedge cluster-randomised controlled trial
Source: Lancet. 2019 May 4;393(10183):1807–18. doi: 10.1016/S0140-6736(18)33212-4 (PMC6497988; doi:10.1016/S0140-6736(18)33212-4)
Supplement: Supplementary appendix [file mmc1.pdf]

THE LANCET

Supplementary appendix

This appendix formed part of the original submission and has been peer reviewed. We post it as supplied by the authors.

Supplement to: Duhig KE, Myers J, Seed PT, et al. Placental growth factor testing to assess women with suspected pre-eclampsia: a multicentre, pragmatic, stepped-wedge cluster-randomised controlled trial. *Lancet* 2019; published online April 1. http://dx.doi.org/10.1016/S0140-6736(18)33212-4.

Supplementary Appendix

Contents

List of Investigators 2

Non-clinical Investigators 2

Trial Definitions 3

Supplementary Tables 4

Supplementary Figures 9

References 10

List of Investigators

Derek Tuffnell and Jennifer Syson, Bradford Teaching Hospitals NHS Foundation Trust

Jenny Myers and Catherine Chmiel, Central Manchester University Hospitals NHS Foundation Trust

Lucy Chappell, Andrew Shennan, Kate Harding, Kate Duhig, Jessica Lowe and Jenie Fetherston, Guy's and St Thomas' NHS Foundation Trust

Gabrielle Bambridge and Amisha Chauhan, Kingston Hospital NHS Foundation Trust

Nigel Simpson and Rebecca Hudson, Leeds Teaching Hospitals NHS Trust

Andrew Sharp and Michelle Dower, Liverpool Women's NHS Foundation Trust

Sonia Barnfield and Mary Alvarez, North Bristol NHS Trust

Asma Khalil and Emily Marler, St George's University Hospitals NHS Foundation Trust

Jo Ficquet and Mel Rich, Royal United Hospitals Bath NHS Foundation Trust

Rachna Bahl and Carole Shahin, University Hospitals Bristol NHS Foundation Trust

Joanna Girling and Bernadette Tilley, Chelsea and Westminster University Hospitals NHS Foundation Trust

Non-clinical Investigators

Carolyn Gill, King's College London

Trial Definitions

Definition of preeclampsia (by the International Society for the Study of Hypertension in Pregnancy 2014 statement).[1]

New onset hypertension (≥ 140 mmHg systolic, or ≥ 90 mmHg diastolic) or worsening of existing hypertension with the coexistence of one or more of the following new-onset conditions: proteinuria (urine protein creatinine ratio of ≥ 30 mg/mmol, renal insufficiency (creatinine ≥ 90umol/L), liver involvement (elevated transaminases at least twice the upper limit of normal), neurological complications (eclampsia, blindness, hyperreflexia with clonus, severe headaches, persistent visual scotomata), haematological complications (platelet count ≤ 150 x 10^9 per litre, disseminated intravascular coagulation, haemolysis), evidence of uteroplacental dysfunction with fetal growth restriction.

Supplementary Tables

Table S1

Recruitment by centre

**Centre**	**Number Recruited**
Bradford Teaching Hospitals NHS Foundation Trust	50 (4.8%)
Central Manchester University Hospitals NHS Foundation Trust	126 (12.2%)
Guy’s and St Thomas’ NHS Foundation Trust	178 (17.2%)
Kingston Hospital NHS Foundation Trust	93 (9.0%)
Leeds Teaching Hospitals NHS Trust	102 (9.9%)
Liverpool Women’s NHS Foundation Trust	116 (11.2%)
North Bristol NHS Trust	67 (6.5%)
St George’s University Hospitals NHS Foundation Trust	79 (7.6%)
Royal United Hospitals Bath NHS Foundation Trust	50 (4.8%)
University Hospitals Bristol NHS Foundation Trust	84 (8.1%)
Chelsea and Westminster University Hospitals NHS Foundation Trust	90 (8.7%)
**Total**	**1035**

Table S2

Secondary Maternal Outcomes

	**Revealed (intervention)** N = 573	**Usual Care (non-intervention)** N = 446	
**Maternal fullPIERS* adverse outcomes, n (%) (non-exclusive)**	22 (3.8%)	24 (5.4%)	Odds ratio 0.32 (0.11-0.96)
Maternal Death	0 (0.0%)	0 (0.0%)	
Central nervous system n (%)			
Eclampsia	0 (0.0%)	2 (0.4%)	
Glasgow coma scale score < 13	0 (0.0%)	0 (0.0%)	
Stroke	0 (0.0%)	2 (0.4%)	
Transient Ischaemic Attack	0 (0.0%)	0 (0.0%)	
Cortical blindness or retinal detachment	0 (0.0%)	0 (0.0%)	
Posterior reversible encephalopathy	0 (0.0%)	0 (0.0%)	
Cardiovascular/ respiratory n (%)			
Positive inotropic support	0 (0.0%)	0 (0.0%)	
Infusion of third parenteral antihypertensive	1 (0.2%)	3 (0.7%)	
Myocardial infarction	0 (0.0%)	1 (0.2%)	
Blood oxygen saturation <90%	1 (0.2%)	1 (0.2%)	
50% oxygen administered >1 hour	0 (0.0%)	0 (0.0%)	
Intubation (other than for caesarean section)	0 (0.0%)	1 (0.2%)	
Pulmonary oedema	2 (0.3%)	0 (0.0%)	
Haematological n (%)			
Transfusion of blood products	9 (1.6%)	14 (3.1%)	
Platelets <50×10⁹/L	4 (0.7%)	4 (0.9%)	
Hepatic n (%)			
Dysfunction**	1 (0.2%)	0 (0.0%)	
Haematoma or rupture	0 (0.0%)	0 (0.0%)	
Renal n (%)			
Severe Acute Kidney Injury***	7 (1.2%)	6 (1.3%)	
Dialysis	0 (0.0%)	1 (0.2%)	
Other adverse events n (%)			
Placental Abruption	4 (0.7%)	5 (1.1%)	

	Revealed (intervention) N = 573	Usual Care (non-intervention) N = 446	
**Time to diagnosis 0-23.9 hours n (%)**	52 (20·3%)	31 (15·8%)	Odds ratio 3·6 (1·16-11·2)
**Gestation at preeclampsia diagnosis (weeks)**<br>Mean (SD)	33·7 (3·6)	34·6 (3·4)	
**Pre-eclampsia diagnosed within 4 weeks of trial entry n (%)**	186 (90·1%)	133 (85·8%)	
**Fetal growth abnormalities on ultrasound (non-exclusive) n (%)**			
Any of the following:			Odds ratio
Scanned	438 (76·6%)	307 (69·3%)	-
Any growth abnormality identified	142 (32·4%)	67 (21·8%)	1·74 (0·87 to 3·47)
Abdominal circumference <10th centile	86 (19·6%)	41 (13·4%)	-
Estimated fetal weight <10th centile	117 (26·7%)	62 (20·2%)	1·49 (0·70 to 3·15)
Umbilical artery pulsatility index >95th	66 (15·1%)	27 (8·8%)	2·94 (1·07 to 8·11)
Absent or reversed end diastolic flow	43 (9·8%)	16 (5·2%)	-
Amniotic fluid index <5th centile	28 (6·4%)	15 (4·9%)	-
**Use of magnesium sulfate n (%)**	72 (12·6%)	64 (14·3%)	Odds ratio 0·95 (0·46 to 1·95)
**Use of antenatal corticosteroids for fetal lung maturity n (%)**	200 (34·9%)	132 (29·6%)	Odds ratio 1·26 (0·75 to 2·11)
**Gestation at delivery, weeks**<br>Mean (SD)	36·6 (3·03)	36·8 (3·03)	Mean difference -0·52 (-0·63 to 0·73)
**Time to delivery (all diagnoses), days**<br>**Geometric mean (SD)**	19·0 (3·1)	17·8 (3·1)	Ratio of means 1·10 (0·99-1·24)

***** as defined in fullPIERS consensus[2].

*** INR >1.2 in the absence of DIC or treatment with Warfarin (DIC is defined as having both: abnormal bleeding and consumptive coagulopathy [i.e., low platelets, abnormal peripheral blood film, or one or more of the following: increased INR, increased APTT, low fibrinogen, increased fibrin degradation products that are outside normal non-pregnancy ranges])

*** Defined as creatinine >150 µmol/L, or >200 µmol/L in women with chronic kidney disease

Table S3

Secondary Perinatal Outcomes

**Perinatal adverse outcome*** **Non-exclusive n (%)**	86 (15.0%)	63 (14.1%)	Odds ratio 1.45 (0.73-2.90)
Central nervous system:			
Intraventricular haemorrhage	7 (1.3%)	11 (2.5%)	
Seizure (any grade)	0 (0.0%)	2 (0.4%)	
Retinopathy of prematurity (any grade)	9 (1.6%)	9 (2.1%)	
Respiratory:			
Respiratory distress syndrome	78 (14.2%)	54 (12.2%)	
Bronchopulmonary dysplasia	5 (0.9%)	3 (0.7%)	
Gastrointestinal:			
Necrotising enterocolitis (stage 2 or 3)	7 (1.2%)	7 (1.6%)	
Perinatal death** n (%)	6 (1.0%)	4 (0.9%)	
Late neonatal death*** (%)	3 (0.5%)	1 (0.2%)	
**Birthweight < 10th centile**	124 (21·8%)	98 (22·1%)	Odds ratio 0·82 (0·46 to 1·44)
**Birthweight < 3rd centile**	58 (10·2%)	43 (9·7%)	Odds ratio 0·89 (0·40 to 2·00)
**Apgar <7 at 5 minutes**	34 (6.0%)	22 (5.2%)	

*Composite of intraventricular haemorrhage (any grade), seizures, retinopathy of prematurity (any grade), respiratory distress syndrome, bronchopulmonary dysplasia, necrotising enterocolitis (stage 2 or 3), perinatal death and late neonatal death.

** Defined as stillbirths from 24 weeks' gestation to deaths up to seven completed days after birth

*** Death between 8 and 27 completed days of life

Table S4

Test performance statistics in women allocated to usual care (concealed testing) for low Placental Growth Factor in prediction of preeclampsia

	**Enrolled <35 weeks' gestation N=265**	**Enrolled 35-36^{+6} weeks' gestation N= 170**
	Preeclampsia requiring delivery within 14 days	Preeclampsia requiring delivery before 37 weeks
**PlGF <100 pg/mL**		
Sensitivity (%; 95% CI)<br>n/N	94·9 (82·7 to 99·4)<br>37/39	96·3 (81·0 to 99·9)<br>25/27
Specificity (%; 95% CI)<br>n/N	52·7 (45·9 to 59·3)<br>119/226	23·8 (17·1 to 31·6)<br>34/143
Positive predictive value (%; 95% CI)<br>n/N	25·7 (18·8 to 33·6)<br>37/144	19·3 (13·0 to 26·9)<br>26/135
Negative predictive value (%; 95% CI)<br>n/N	98·3 (94·2 to 99·8)<br>119/121	97·1 (85·1 to 99·9)<br>34/35
Positive likelihood ratio (95% CI)	2·00 (1·71 to 2·34)	1·26 (1·12 to 1·42)
Negative likelihood ratio (95% CI)	0·10 (0·03 to 0·38)	0·16 (0·02 to 1·09)
**PlGF <12 pg/mL**		
Sensitivity (%; 95% CI)<br>n/N	74·4 (57·9 to 87·0)<br>29/39	37·0 (19·4 to 57·6)<br>10/27
Specificity (%; 95% CI)<br>n/N	84·1 (78·6 to 88·6)<br>190/226	78·3 (70·7 to 84·8)<br>12/143
Positive predictive value (%; 95% CI)<br>n/N	44·6 (32·3 to 57·5)<br>29/65	24·4 (12·4 to 40·3)<br>10/41
Negative predictive value (%; 95% CI)<br>n/N	95·0 (91·0 to 97·6)<br>190/200	86·8 (79·7 to 92·1)<br>112/129
Positive likelihood ratio (95% CI)	4·67 (3·28 to 6·64)	1·71 (0·95 to 3·06)
Negative likelihood ratio (95% CI)	0·30 (0·18 to 0·52)	0·80 (0·59 to 1·09)

Supplementary Figures

Figure S1

Clinical Management Algorithm

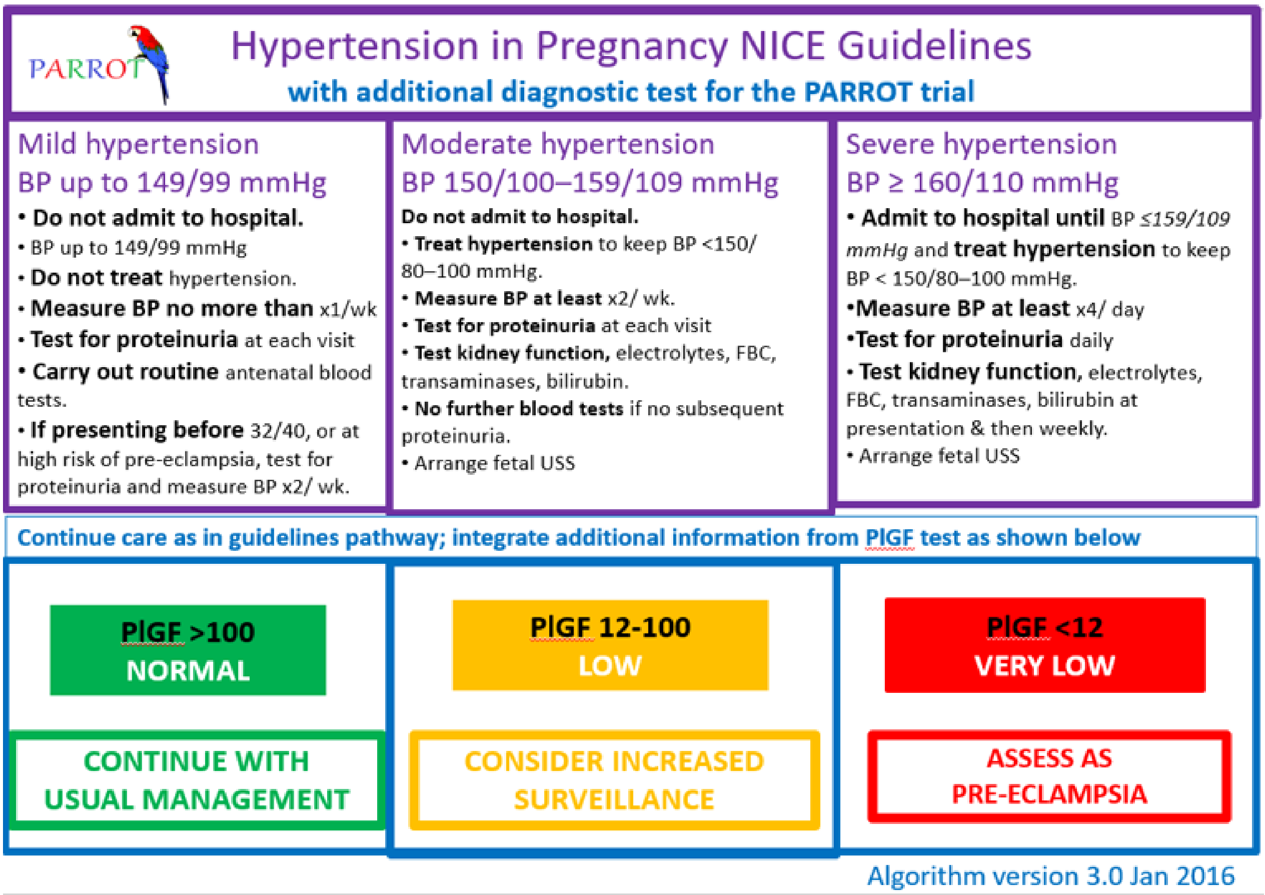

References

1. Tranquilli AL, Dekker G, Magee L, et al. The classification, diagnosis and management of the hypertensive disorders of pregnancy: A revised statement from the ISSHP. Pregnancy hypertension 2014;4:97-104.

2. von Dadelszen P, Payne B, Li J, et al. Prediction of adverse maternal outcomes in pre-eclampsia: development and validation of the fullPIERS model. Lancet (London, England) 2011;377:219-27.
